# Online information on medical cannabis is not always aligned with scientific evidence and may raise unrealistic expectations

**DOI:** 10.1186/s42238-022-00145-w

**Published:** 2022-07-11

**Authors:** Arthur Cassa Macedo, André Oliveira Vilela de Faria, Isabella Bizzi, Fabrício A. Moreira, Alessandro Colasanti, Pietro Ghezzi

**Affiliations:** 1 Universidade Federal de Minas Gerais, Belo Horizonte, Minas Gerais Brazil; 2 Universidade Federal do Rio Grande do Sul, Porto Alegre, Rio Grande do Sul Brazil; 3 Brighton & Sussex Medical School, Trafford Centre, University of Sussex, Falmer, Brighton, East Sussex BN1 9RY UK; 4 Università di Urbino, Urbino, Italy

**Keywords:** Cannabis, Health information, Websites, Consumer health information

## Abstract

**Background:**

There is a growing literature on the potential medical uses of *Cannabis sativa* and cannabinoid compounds. Although these have only been approved by regulatory agencies for a few indications, there is a hype about their possible benefits in a variety of conditions and a large market in the wellness industry. As in many cases patients search for information on cannabis products online, we have analyzed the information on medical cannabis available on the Internet. Therefore, this study aims at assessing the quality of the information available online on medical cannabis.

**Methods:**

We searched “medical cannabis” on June 2019 using google.com and downloaded the first 243 websites. After excluding dead links or websites with no information about cannabis, 176 websites were included. They were then classified for their typology (e.g., commercial, government, news outlets). As an indicator of trustworthiness, we used the Journal of American Medical Association (JAMA) score, which assesses the indication of date, author, ownership of the website, and the presence of references. We also considered if a website is certified by Health-On-the-Net (HON), an independent organization, by displaying a HONCode symbol. Subsequently, we performed a content analysis to assess both the medical cannabis indications mentioned by webpages and the completeness of the information provided (whether they mentioned potential side effects and legal/regulatory issues or not).

**Results:**

Analyzing 176 webpages returned by a search engine, we found that 52% of them were news websites. Pain, epilepsy, and multiple sclerosis were the most frequently mentioned therapeutic areas (cited in 92, 84 and 80 webpages, respectively), which did not always match those for which there is regulatory approval. Information was also incomplete, with only 22% of the webpages mentioning potential side effects. Health portal websites provided the most complete information, with all of them (*n* = 7) reporting side effects. On average, 80% of webpages had a neutral stance on the potential benefits of medical cannabis, with commercial websites having more frequently a positive stance (67%).

**Conclusions:**

We conclude that the information that can be found online is not always aligned in terms of the therapeutic areas for which science-based evidence is often still weak.

**Supplementary Information:**

The online version contains supplementary material available at 10.1186/s42238-022-00145-w.

## Background

There is growing literature on the potential medical use of *Cannabis sativa* and its derived molecules, termed cannabinoids, including Δ^9^-tetrahydrocannabinol (THC) and cannabidiol (CBD) (World Health Organization Expert Committee on Drug Dependence, 2018). The available pharmaceutical preparations comprise plant-derived THC, synthetic THC (dronabinol), the synthetic cannabinoid nabilone, plant-derived CBD, and a combination of plant-derived THC and CBD. Despite the recent growth in interest, and suggestions that the earliest medical use of cannabis products could be traced back to ancient times (Zias et al., 1993), at present, only very few cannabis-based medicines have been approved for clinical use. In some countries, a plant extract containing a combination of approximately equal parts of THC and CBD is in clinical use for alleviating the symptoms of multiple sclerosis. Moreover, a CBD-containing extract has been approved for the treatment of refractory epileptic syndromes, such as Lennox-Gastaut syndrome or Dravet syndrome, and low-potency synthetic cannabinoids can be prescribed for nausea associated with cancer chemotherapy and for the treatment of anorexia associated with weight loss in acquired immune deficiency syndrome (AIDS) patients (Black et al., 2019) (Food and Drug Administration, 2019). Cannabis-derived products have complex pharmacological characteristics, such as the opposing pharmacological and behavioral effects of the two main constituents of cannabis (Morgan et al., 2010), further complicated by the high variability of cannabis products in terms of their pharmacodynamics and kinetics features, as well as their delivery through various routes of administration.

In addition to the approved indications for which there is scientific evidence, there is a hype about the use of many cannabis-derived products for a variety of conditions (Eisenstein, 2019; Stith et al., 2019), with the market of CBD in the wellness industry in the United States of America (US) predicted to be at US$24 billion by 2023 (Giammona & Einhorn, 2019). The previous history of cannabis as a recreational drug could also potentially lead to a polarized view on its medical use, with either uncritical support independent of scientific evidence or a negative bias. In this context, the public will often gather non-specialized information on the Web rather than seeking advice from their doctors.

A 2017 survey has shown that 38.5% of US adults have searched for health information online (Finney Rutten et al., 2019), and the quality of the information available has been an active area of research. It is therefore expected that, with the development of medicinal products based on cannabis and their legalization in many countries, many patients will look online for information on their efficacy and availability (Kruger et al., 2020; Kruger et al., 2021).

Recent studies have analyzed the information available online on medical cannabis in general (Kruger et al., 2020) or for specific indications such as glaucoma. (Jia et al., 2021) A study by Jia et al. found that 24% of the webpages returned by Google (and 59% of YouTube videos) had a positive stance on the use of medical cannabis in glaucoma (Jia et al., 2021). Ng et al. (Ng et al., 2021), although not specifically investigating the stance of the websites, have shown that they often were of low quality according to standard measures of information quality and that quality was higher for health portals and lower for commercial websites.

We have observed that often online information does not match scientific evidence, potentially pointing the public to the use of health supplements for indications for which there is no high-quality evidence, as in the case of probiotics or antioxidant supplements (Aslam et al., 2017; Neunez et al., 2020). Cochrane reviews also showed that cannabinoids use is associated with an increased risk of transient adverse events including weakness, dizziness, sleepiness, difficulty with concentration, memory loss, confusion, headache, nausea, and fatigue (Kafil et al., 2018a; Kafil et al., 2018b; Smith et al., 2015), and, for completeness, information should also describe these side effects.

The present study aims at assessing the information available online on medical cannabis both in terms of information quality criteria and content, particularly the therapeutic area mentioned and the completeness of the information. The frequency with which specific indications are mentioned in the analyzed webpages is discussed in the context of the evidence available on Cochrane and the assessment of the evidence strength. We also referred to the level of evidence for the use of medical cannabis products in the report of the US National Academy of Sciences (National Academies of Sciences, Engineering, and Medicine, 2017), to allow reference to the study on online information on cannabis by Kruger et al. (Kruger et al., 2020)

## Methods

We searched “medical cannabis” on google.com in June 2019 and downloaded the first 243 websites. We looked first at the typology of websites, whether professional, commercial, news, or others, and their trustworthiness indicators such as the Journal of the American Medical Association (JAMA) score and the HONCode certification. The JAMA score assesses the presence of four types of information (i.e., whether the website indicates authors, date, references to the source of information provided, and information on the ownership of the website) (Silberg et al., 1997). The HONCode is a certification that websites can request on a voluntary basis from the Health-On-the-Net (HON) foundation, a non-profit organization based in Switzerland that certifies medical and health websites based on aspects related to ethics, transparency, and trustworthiness (Boyer et al., 1998). Of note, neither the JAMA score nor the HONCode evaluate the content in terms of medical accuracy or validity.

We then analyzed the content to find which diseases or indications were mentioned in the webpages and correlated this with the number of clinical trials or reviews for those indications that are available from the Cochrane center. Finally, we considered whether the information was complete, i.e., if webpages mentioned potential side effects and legal/regulatory issues.

The search term “medical cannabis” was used as Google trends showed this as one of the top five topics related to worldwide searches on “cannabis.” Searches were made on google.com from Brighton, United Kingdom (UK), in June 2019, using the URL google.com/ncr, to avoid redirection to the localized version of the search engine, and after deleting cookies and browsing history, to limit personalization of the search results. Based on previous studies, we aimed at collecting a sample of 150–170 webpages. We downloaded the first 243 links. Of these, 67 websites were excluded for the following reasons: eight were referring to or selling a book; 17 were index pages, aggregators, or dynamic pages returning results from a search; two contained no information as they were just lists of doctors or government offices; 14 were inaccessible or blocked websites or dead links; 17 contained no information about medical cannabis; five were about a new degree in medical cannabis opened at the University of Maryland; and three were links to a video. Therefore, 176 webpages were analyzed for their content. The webpages were visited and analyzed using a previously validated methodology, based on intrinsic criteria and content. The classification by website typology has been validated in our previous studies with inter-rater agreement ranging between 83% and 95%, depending on the coders (Ghezzi et al., 2019). Determining the stance of the website about medical cannabis (positive, negative or neutral) was more subjective. On a sample of ten webpages assessed independently by all coders in the present study, the average agreement among three raters (ACM, AOVdF, and IB) was 61%. Disagreements were resolved by discussion via email, with one of us (PG) overseeing the discussion and final decision.

### Intrinsic criteria:

1. Websites were classified as commercial (C), government (G), health portals (HP), news (N), non-profit (NP), or scientific journal (SJ) as described elsewhere (Aslam et al., 2017; Neunez et al., 2020). Websites not belonging to any of these typologies or where it was difficult to establish a typology were classified as “others” (O)
2. JAMA score. A score of 0 to 4 was assigned based on the presence of the following information: author, date, references to the source of information provided, and ownership of the website (Silberg et al., 1997). The presence of each of these criteria was counted as 1; therefore, the JAMA score ranged from 0 to 4
3. HONcode. The HONcode certification was detected by the presence of a valid HONcode seal of approval on the webpage (Boyer et al., 1998)

### Content analysis:

1. Indication. We recorded the disease or biological process for which the use of medical cannabis was mentioned
2. Stance about medical cannabis (in terms of efficacy or use), whether positive, neutral, or negative. This was based on the wording of the text. Examples of classification of stance based on text contained in the webpage are shown in Supplementary File 2
3. We recorded whether the webpage mentioned potential side effects and regulatory/legal issues associated with the use of cannabis products

Statistical analysis was performed using GraphPad Prism v.9. The raw data and list of webpages are provided in Supplementary File 1.

## Results

### Type of websites

**Fig. 1:**
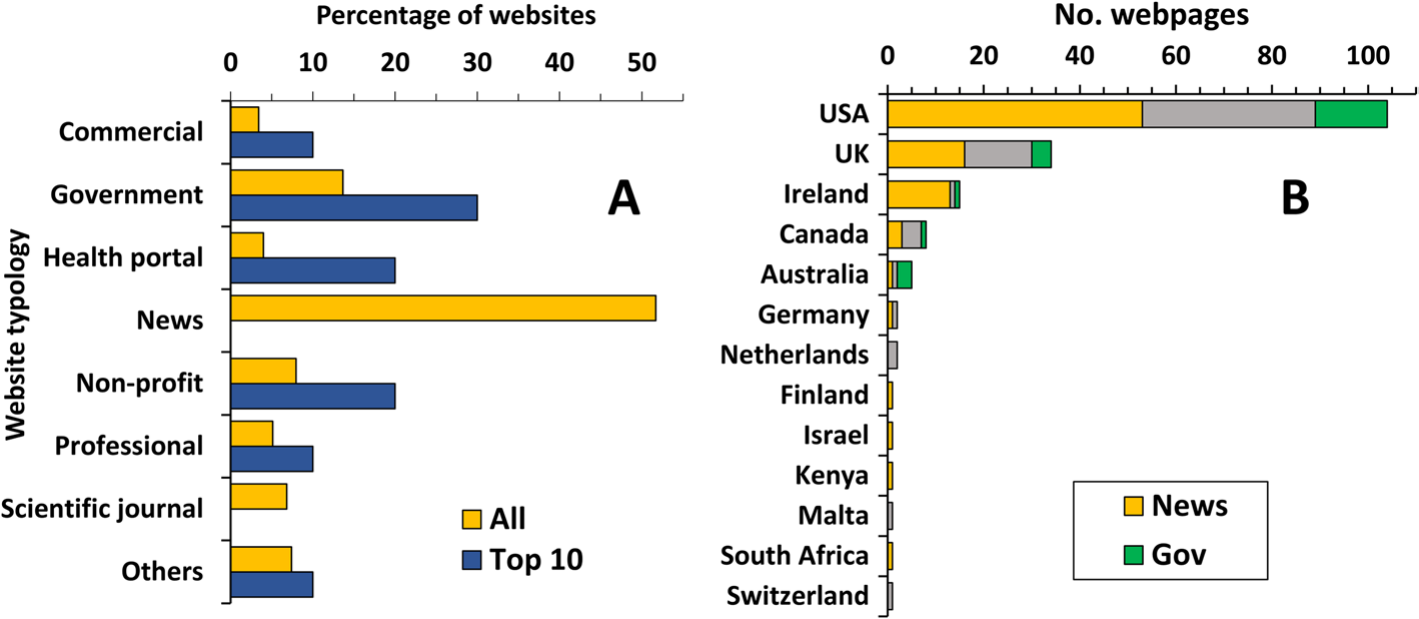
Search trends in Google for “cannabis” and “medical cannabis.” Numbers represent search interest relative to the highest point on the chart for the given region and time for each search term. A value of 100 is the peak popularity for the term. A value of 50 means that the term is half as popular. A score of 0 means there was not enough data for this term. **A** Typologies of websites returned by Google. Data represent the percentage of websites across the whole search and the top 10 webpages in the Google search results. C, commercial; G, government; HP, health portals; N, news; NP, no profit; P, professional; SJ, scientific journal; O, other (unclassified). **B** Number of websites returned per country of origin. For each nation, news are in orange, government websites in green, and all other typologies in grey.

Figure 1A shows the website typologies in the whole search engine results page (SERP; 176 webpages) and in the top ten returned by Google. In the whole SERP, the most frequent typology was represented by websites from news outlets (52%) followed by government websites (14%). No news websites, however, were present in the top ten results, while a higher ranking was given by Google to websites from non-profit organizations (20% in the top ten vs. 8% in the whole SERP), health portals (20% vs. 4%), and government websites (30% vs. 14%). When comparing the frequency of each typology in the top ten results versus the rest of the SERP, only news websites were significantly under-represented (Fisher’s test followed by adjustment for eight multiple comparisons using the two-stage linear step-up procedure of Benjamini, Krieger, and Yekutieli set at a false discovery rate of 5%). As shown in Fig. 1B, 59% of the websites were from the USA, 19% from the UK, 9% from Ireland, and a small number from other countries.

### Trustworthiness indicators

Overall, the 176 pages had a median JAMA score of 3, interquartile range (IQR) 1, min 0, max 4. However, the JAMA score differed significantly across the different typologies of websites, with health portals and scientific journals scoring the highest (respectively: median 4, IQR 1 and median 4, IQR 0). The median JAMA scores of commercial and government websites were 1, IQR 2 and median 1, IQR 1, respectively; that of non-profit organizations was 2, IQR 0.5. Commercial, government, and non-profit organizations webpages scored significantly lower when compared with the rest of the SERP (Mann-Whitney test followed by adjustment for eight multiple comparisons using the two-stage linear step-up procedure of Benjamini, Krieger, and Yekutieli set at a false discovery rate of 5%).

Only eight of the 176 websites had a HONcode certification, four of which were health portals, meaning 57% of this type of websites were HON-certified. Three of the top ten websites (30%) had a HONcode, significantly more than the 3% (5/166) in the remaining websites (*P* = 0.0064 by a two-tailed Fisher’s test).

### Content analysis: diseases and conditions mentioned

As mentioned earlier, cannabis-derived products are approved for a limited number of indications. However, the webpages analyzed mentioned many more diseases and conditions in relation to the possible benefits of medical cannabis. In this respect, we wondered whether the frequency with which these conditions are ranked reflects, if not the approval by regulatory agencies, at least the amount of clinical research. As a proxy for the clinical research activity on medical cannabis, we searched the Cochrane library on November 22, 2019, and considered the numbers of randomized placebo-controlled clinical trials (RCT) and of Cochrane reviews in the database. The same figure also reports, as a color code, the conclusions on the level of evidence for the use of medical cannabis products in the report by the US National Academies of Sciences, Engineering, and Medicine (Kruger et al., 2020; National Academies of Sciences, Engineering, and Medicine, 2017), as indicated in the legend.

**Fig. 2:**
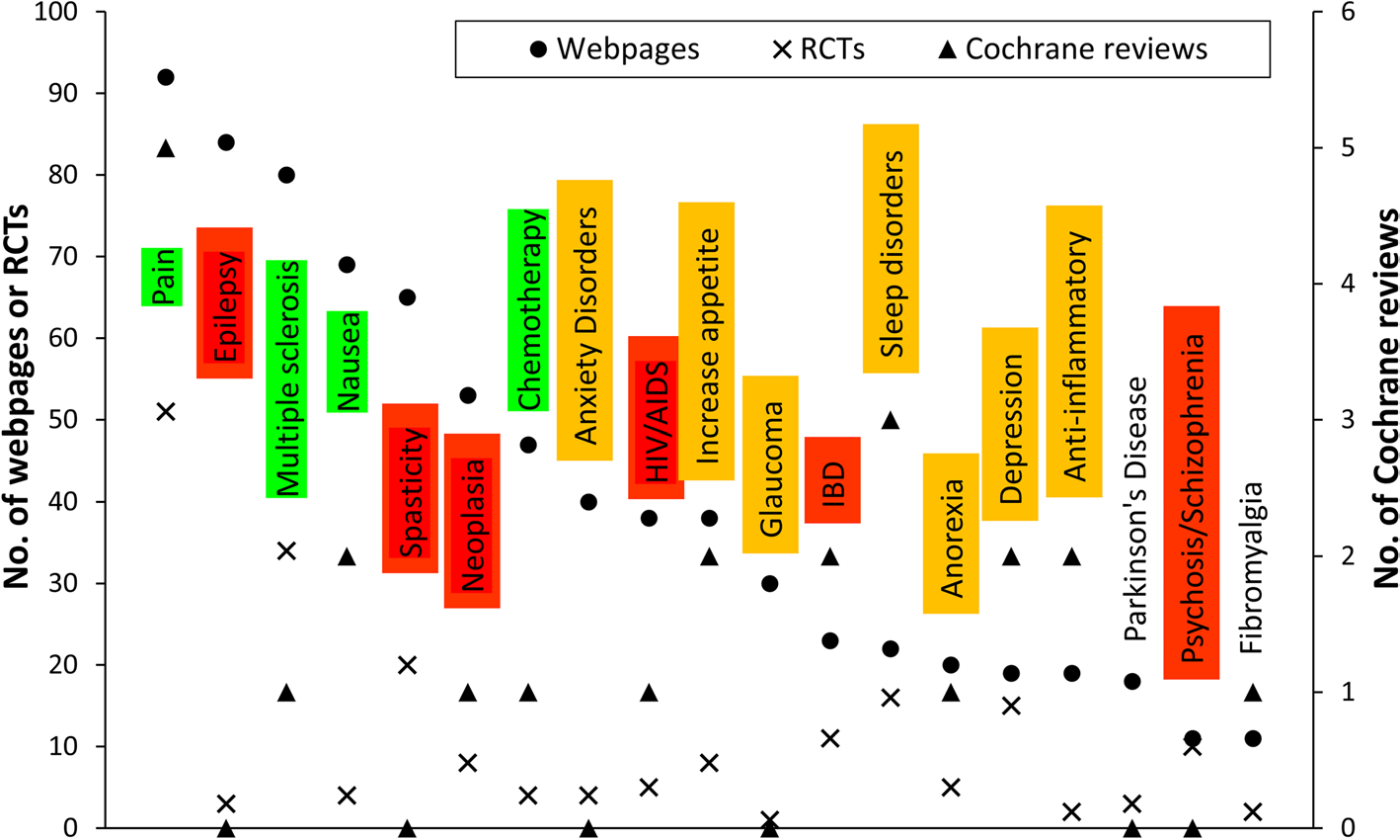
Therapeutic areas mentioned in the webpage (solid circles) in relation to the number of Cochrane reviews (triangles) and randomized clinical trials, RCTs, (x) in the Cochrane database for that indication. Colors indicate the conclusions on the level of evidence by the National Academies of Sciences, Engineering, and Medicine (NASEM): green, conclusive or substantial; yellow, limited; red, none or insufficient. No color indicates that NASEM did not report a conclusion on that indication. Notes: the conclusive or substantial evidence for pain is limited to chronic pain specifically (Kruger et al., 2020)

The results are shown in Fig. 2. It can be seen that the indications most frequently mentioned by webpages are pain, epilepsy, and multiple sclerosis. It can be noted that many indications are mentioned by a significant number of webpages despite the relatively small number of RCTs. In general, webpages mention a large number of conditions for which medical cannabis could have benefits, far more than those indications for which these products have been approved by regulatory agencies.

**Fig. 3:**
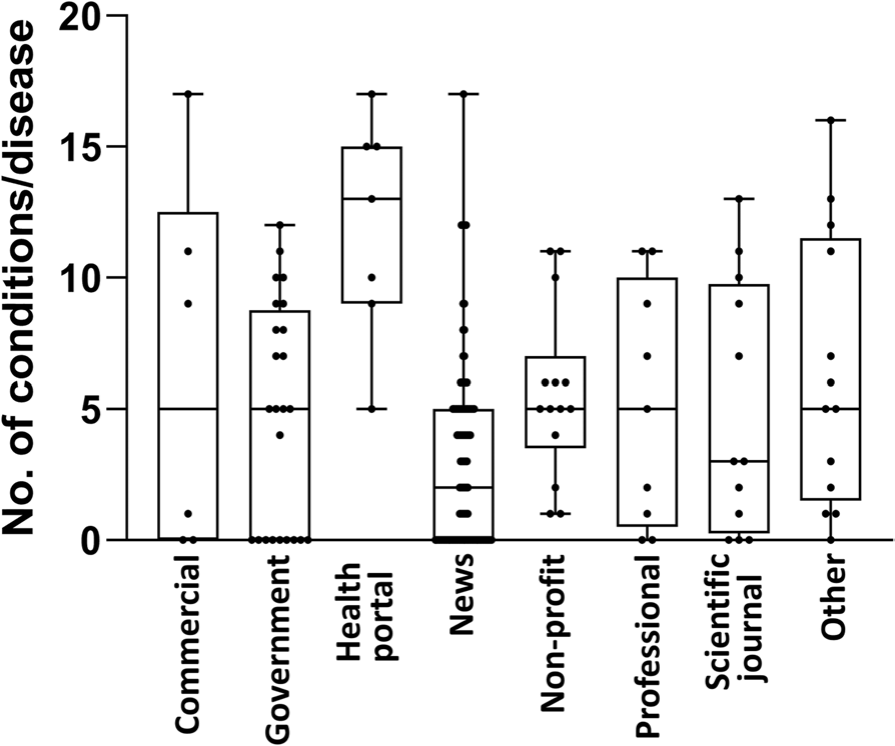
Number of indications mentioned by webpages of different typologies. Data represent the number of conditions mentioned as median, IQR (interquartile range), minimum, maximum. C, commercial; G, government; HP, health portals; N, news; NP, no profit; P, professional; SJ, scientific journal; O, other (unclassified)

There was a difference in the number of diseases mentioned across different types of websites. As shown in Fig. 3, health portals mentioned the largest number of diseases (median, 13; IQR, 9.15) and news the lowest (median, 2; IQR 0.5). Both values were significantly different from the rest of the SERP (Mann-Whitney test followed by adjustment for eight multiple comparisons using the two-stage linear step-up procedure of Benjamini, Krieger, and Yekutieli set at a false discovery rate of 5%).

### Completeness of information

We also assessed whether webpages report potential side effects and legal/regulatory issues of medical cannabis products. Side effects were mentioned by only 22% of webpages and legal/regulatory aspects by 82% of them. However, there were differences in the completeness of the information provided across typologies, particularly for side effects. As shown in Table 1, all health portals mentioned the side effects of medical cannabis, a frequency that was significantly higher when compared with the remaining 169 webpages; side effects were also more frequently mentioned by websites from non-profit organizations. By contrast, only 5 of the 91 news websites mentioned side effects, significantly less than the remaining 85 webpages in the search. There were no significant differences in the frequency of mentions of regulatory aspects.

**Table 1:** Mention of side effects and legal/regulatory aspects across different website typologies

Typology	Side effects	Legal/regulatory
Commercial	17% (1/6)	50% (3/6)
Government	25% (6/24)	92% (22/24)
Health portals	100% (7/7)^a^	71% (5/7)
News	5% (5/91)^a^	84% (76/91)
Non-profit	57% (8/14)^a^	71% (10/14)
Professional	44% (4/9)	67% (6/9)
Scientific journal	33% (4/12)	58% (7/12)
Others	31% (4/13)	85% (11/13)

^a^Significantly different vs the rest of the SERP by a two-tailed Fisher’s test followed by adjustment for eight multiple comparisons using the two-stage linear step-up procedure of Benjamini, Krieger, and Yekutieli set at a false discovery rate of 5%

### Stance towards medical cannabis

**Fig. 4:**
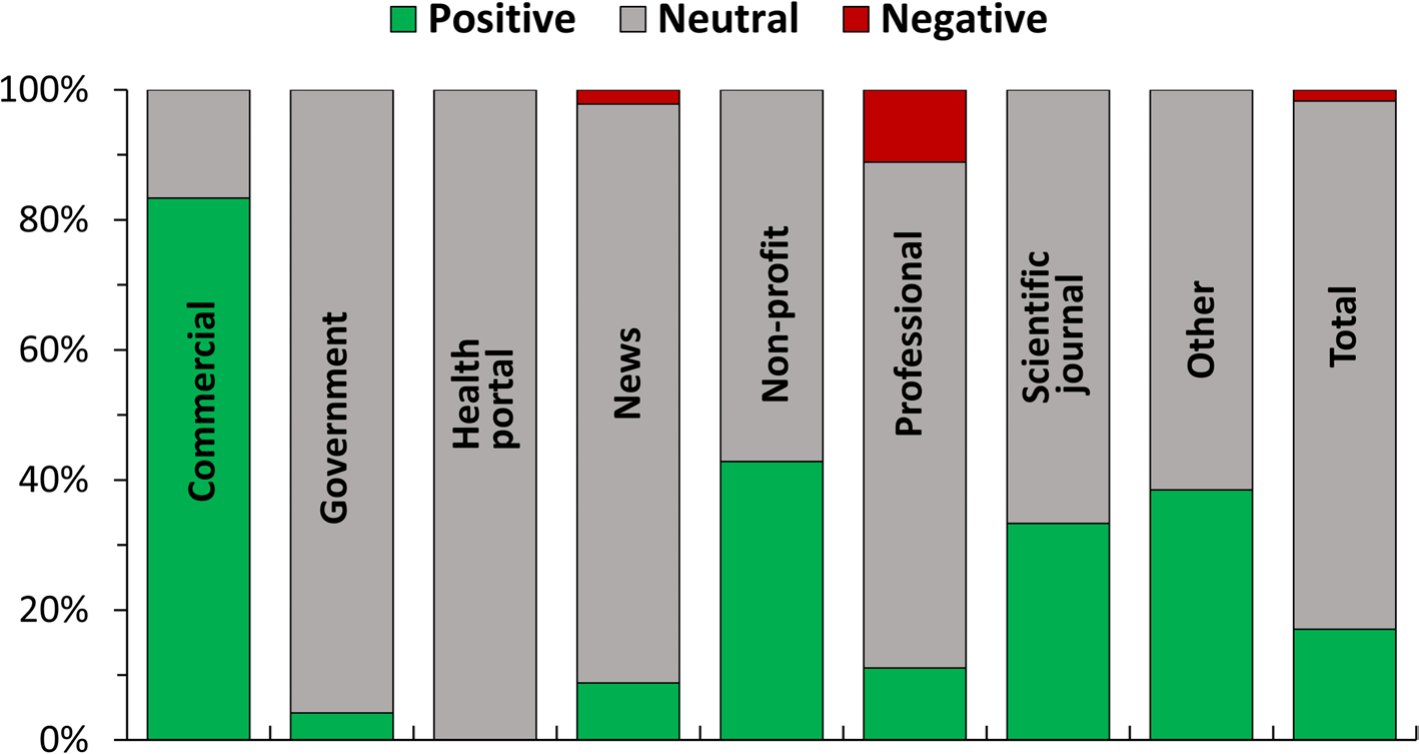
Stance toward medical cannabis in different typologies of websites. Data represent the percentage of webpages with a specific stance on cannabis. C, commercial; G, government; HP, health portals; N, news; NP, no profit; P, professional; SJ, scientific journal; O, other (unclassified)

The majority (81%) of the webpages had a stance towards medical cannabis which we defined as neutral, 17% positive and only 2% negative. A sub-analysis in Fig. 4 shows differences among the typologies of websites. The highest proportion of positive pages (5 out of 6, 83%) was observed in commercial websites, followed by non-profit; both frequencies were significantly higher when compared with the rest of the SERP (two-tailed Fisher’s test followed by adjustment for eight multiple comparisons using the two-stage linear step-up procedure of Benjamini, Krieger, and Yekutieli set at a false discovery rate of 5%). Government and news websites had the lowest frequency of pages with a positive stance, which was significantly different only in the case of news. All pages from health portals and 88% of the news websites had a neutral view. Only four webpages had a negative stance on medical cannabis.

## Discussion

This study shows that over half of the webpages containing information about medical cannabis are from news websites, which indicates the newsworthiness of this topic. It should be mentioned, however, that the ranking made by Google prioritizes government websites, those from non-profit organizations and health portals over news outlets. Websites bearing the HONcode, an independent health information quality certification, were also ranked significantly higher, in agreement with our previous findings on information online about probiotics (Neunez et al., 2020), confirming the observation that Google uses effective criteria to prioritize high-quality information.

Content analysis in terms of disease/indications showed a mismatch between the therapeutic areas mentioned on the Web and those for which there is regulatory approval. The therapeutic area most frequently mentioned on the Web is pain. In the UK, NICE (The National Institute for Health and Care Excellence) guidelines specifically recommend not to prescribe cannabis products for chronic pain unless as part of a clinical trial (NICE, 2019) and pain treatment is not an approved indication for any cannabis product in the US as of February 2020 (FDA, 2020).

The second most frequent indication mentioned on the Web is epilepsy. It should be noted, however, that the only cannabis-based medicine approved for this indication, plant-derived CBD, is licensed exclusively for the treatment of specific epileptic syndromes, namely Dravet and Lennox-Gastaut Syndromes (Friedman & Devinsky, 2015).

Another indication frequently mentioned is multiple sclerosis, despite the limited approval of cannabis-derived products for this disease. So far, an oro-mucosal spray containing plant-derived THC and CBD (nabiximols) is only approved, for instance in the UK, for the treatment of multiple sclerosis-associated spasticity.

A number of webpages mention cancer and chemotherapy. In fact, there is moderate evidence that THC, dronabinol and nabilone may be useful for treating refractory chemotherapy-induced nausea and vomiting, although they failed to show superiority as compared to conventional drugs, particularly prochlorperazine (Smith et al., 2015). In patients with human immunodeficiency virus (HIV)/AIDS, the studies have been of short duration and limited to a small number of patients, preventing any solid conclusion of efficacy (Lutge et al., 2013).

Finally, scarce evidence suggests the CBD might be useful for the treatment of other neurological and psychiatric disorders, such as schizophrenia and anxiety (Black et al., 2019). Although there is little clinical evidence for these indications (Crippa et al., 2018; Rohleder et al., 2016), anxiety is mentioned by a significant number of webpages.

Our research complements a recent study by Kruger et al. (Kruger et al., 2020) that analyzed the information online on medical cannabis. Despite using different search terms in Google, the authors reported, like us, that pain, epilepsy, nausea, and multiple sclerosis are the medical conditions most frequently described online (Kruger et al., 2020). We also found a percentage of webpages reporting potential side effects, 22%, similar to what reported by Kruger (20%). However, our sub-analysis by website typology (reported in Table 1) identified a large variability, with side effects mentioned more frequently by health portals and non-profit organizations and much less frequently by commercial websites and news outlets, thus suggesting a bias associated with commercial interests and newsworthiness. Poor reporting on the risks associated with medical cannabis was also observed in studies on print news outlets in California (Halvorson et al., 2018) and in Canadian news media (Gunning & Illes, 2021).

Therefore, the general picture is that there is a partial mismatch between the indications mentioned on the Web for cannabis-based products and the regulatory approval, particularly for the treatment of pain. Often, an indication is frequently mentioned on the web despite there being few RCTs listed in the Cochrane database, and this is, for instance, the case of nausea, cancer, and anxiety disorders. There is also a mismatch between the frequency in which an indication is mentioned on websites and the level of evidence as defined by the NASEM report.

This breadth of online information might potentially raise the interest of the public in the use of medical cannabis for a range of indications that is broader than that of indications that are actually approved, potentially making it more attractive for the public to use cannabis supplements as self-medication in absence of a medical prescription.

In addition, there might be a misconception regarding the safety of cannabis-based products. As a result, self-initiated use may lead to side effects, drug interactions, use despite contraindications, and non-adherence to medical treatments. Among the reported side effects of cannabis-based products are nausea, vomiting, sedation, and motor impairment (Arnold, 2021; Bar-Lev Schleider et al., 2018). As for the drug interactions, cannabinoids may interfere with the effects of various psychoactive drugs in different ways, especially after long-term use (Gottschling et al., 2020). Contraindications for the use of certain cannabis-based products include brain disorders, particularly those with psychotic features, such as schizophrenia and bipolar disorder (Gottschling et al., 2020). Finally, self-medication may lead to poor adherence to doctor-oriented treatments, interfering with therapeutic effectiveness (Osterberg & Blaschke, 2005).

On the other hand, we found that the large majority of websites (88%) had a neutral stance on the use of cannabis, indicating that the information available online is not particularly polarized. One exception is represented by commercial websites that have a largely positive stance (67%), which can be explained by the fact that commercial websites are often selling cannabis-derived products.

Another aspect of health information online is that of the completeness of the information provided, which was suggested as a criterion for health information quality (Dutta-Bergman, 2004). In two recent studies, we noted that a key aspect of completeness of health information is the mention of potential side effects and regulatory issues (Neunez et al., 2020; Manley & Ghezzi, 2018). In the present study, while regulatory issues were often mentioned, only 22% of websites mentioned the potential side effects, and this was due, in particular, to the paucity of this type of information in news outlets and commercial websites. The websites providing the most complete information were those from health portals, all of them mentioning the potential adverse effects.

The main limitation of the present study is the protocol through which the sample of webpages was collected, as it may vary with time and with the search query. Although we used generic and neutral search terms, the results could be different when searching for cannabis and a specific health condition. This limitation, however, could have been at least partially circumvented by evaluating a large number of webpages. Another limitation is that we only used Google as a search engine. These may not be a representative sample of the infosphere, because Google recently implemented high-quality standards in the page ranking of what they define as “your money your life” pages. According to these guidelines, the algorithm used by Google gives a higher ranking to pages written by people or organizations with medical expertise, authoritativeness and trustworthiness, and where the information provided is aligned with the scientific consensus on the topic (Google, 2020). As we noted elsewhere, other search engines provide more lower-quality results than Google (Ghezzi et al., 2019). On the other hand, as Google has around 90% of the search engine market share, the websites it returns are the ones that the user would likely find. Not only different search engines might provide different results, but a recent study found that one third of the health-related webpages are present on Facebook (Libert, 2015), which is also used for information seeking (Anita & Williams, 2013).

Another cautionary note is the fact that we refer to the Cochrane database and the NASEM conclusions for assessing the level of evidence for the effectiveness of cannabis products in specific indications. Conclusions on the effectiveness of cannabis products in disease are not within the scope of the present study, and the field is developing rapidly with new results from clinical trials being reported. Furthermore, there are different cannabis products being tested and it is often difficult to compare the evidence obtained for different diseases with different products.

## Conclusions

In conclusion, our study indicates that the information available on the Web could raise unrealistic expectations in the public and contribute to a hype that could potentially lead patients to use cannabis-based products as self-medication when describing potential indications for which there is no strong evidence of efficacy. It would be important for website and news editors to provide references to clinical evidence in terms of RCTs, as well as inform the public of the potential side effects of cannabis-based products.

While it would be desirable that news outlets and commercial websites provided more complete information, our findings suggest that health professionals should point their patients toward websites from non-profit organizations or health portals to get more comprehensive information and allow them to make informed decisions. It is reassuring, in this respect, that our study shows that the ranking provided by Google gives higher visibility to health portals and non-profit organizations, in agreement with the findings by Ng et al. for websites on medical cannabis and chronic pain (Ng et al., 2021). The number of webpages mentioning medical cannabis in the context of specific indications not aligned with regulatory approval might indicate both where public health authorities should focus their strategies to disseminate information and, potentially, where a clear answer from clinical trials or systematic reviews are needed.

## Supplementary Information

**Additional file 1: Table 1.** Examples of stance about cannabis.

**Additional file 2: **Spreadsheet with raw data.

## Data Availability

The raw data and list of webpages are provided in the supplementary file.

## Abbreviations

AIDS: Acquired immune deficiency syndrome
CBD: Cannabidiol
HIV: Human immunodeficiency virus
HON: Health-On-the-Net
IQR: Interquartile range
JAMA: Journal of the American Medical Association
NASEM: National Academies of Sciences, Engineering, and Medicine
NICE: The National Institute for Health and Care Excellence
RCT: Randomized placebo-controlled clinical trials
SERP: Search engine results pages
THC: Δ^9^-Tetrahydrocannabinol
UK: United Kingdom
US: United States of America
