# Supplementary material for: Online information on medical cannabis is not always aligned with scientific evidence and may raise unrealistic expectations
Source: J Cannabis Res. 2022 Jul 11;4:37. doi: 10.1186/s42238-022-00145-w (PMC9277882; doi:10.1186/s42238-022-00145-w)
Supplement: Supplementary file 1 — Additional file 1: Table 1. Examples of stance about cannabis. [file 42238_2022_145_MOESM1_ESM.docx]

**Additional Table 1. Examples of stance about cannabis**

| ***Examples of positive webpages*** |
| --- |
| “Marijuana has been shown to alleviate symptoms of a huge variety of serious medical conditions including cancer, AIDS, and glaucoma, and is often an effective alternative to synthetic painkillers” (<http://www.drugpolicy.org/issues/medical-marijuana>) |
| “Both forms of cannabis, hemp and marijuana, have been shown to contain medically beneficial levels of differing cannabinoids, active compounds found in the cannabis plant” (<https://www.medicalmarijuanainc.com/>) |
| “Our study suggest that Cannabis therapy, as an adjunct a traditional analgesic therapy, can be an efficacious tool to make more effective the management of chronic pain and its consequences on functional and psychological dimension. Further randomized, controlled trials are needed to confirm our conclusions” (<https://www.ncbi.nlm.nih.gov/pubmed/29938740>) |
| “The claims by medical users that cannabis reduces the symptoms of MS has been confirmed by UK government trials” ( <https://www.ukcia.org/medical/index.php>) |
| “However, reviews of published studies have generally shown that synthetic cannabinoids favorably impact symptoms of pain and spasticity” (<https://www.nationalmssociety.org/Treating-MS/Complementary-Alternative-Medicines/Marijuana/Marijuana-FAQs>) |
| ***Examples of negative webpages*** |
| “According to the Department of Health, it is important to note that where a cannabis product is a specified controlled drug legally permitted for medical use, in connection with the MCAP, this does not signify any endorsement whatsoever of the safety, quality or efficacy of the specified controlled drug for the indication prescribed” (<https://www.imt.ie/news/minister-no-liability-access-medical-cannabis-programme-26-06-2019/>) |
| “Medical marijuana dispensing is associated with reduced perception of marijuana-related risks and increased rates of marijuana use among adolescents” (https://www.aacap.org/AACAP/Policy_Statements/2012/AACAP_Medical_Marijuana_Policy_Statement.aspx) |
| ***Examples of neutral webpages*** |
| “While cannabis, or marijuana, has been around for a long time, there is still not much formal evidence for doctors to rely on if they are thinking about prescribing a medicinal cannabis product to a patient. The Commonwealth Government has released documents which summarise the evidence so far that medicinal cannabis may be useful in treating some conditions “(<https://www.betterhealth.vic.gov.au/health/conditionsandtreatments/medicinal-cannabis>) |
| “The use of cannabis as medicine has not been rigorously tested due to production and governmental restrictions, resulting in limited clinical research to define the safety and efficacy of using cannabis to treat diseases. Preliminary evidence suggests that cannabis can reduce nausea and vomiting during chemotherapy, improve appetite in people with HIV/AIDS, reduces chronic pain and muscle spasms and treats severe forms of epilepsy” (<https://en.wikipedia.org/wiki/Medical_cannabis>) |
| “So far, researchers haven't conducted enough large-scale clinical trials that show that the benefits of the marijuana plant (as opposed to its cannabinoid ingredients) outweigh its risks in patients it's meant to treat” <https://www.drugabuse.gov/publications/drugfacts/marijuana-medicine>) |
| “There haven’t been any studies comparing medical cannabis with other medicines already licensed for treating epilepsy. So we don’t know if medical cannabis is more or less effective than other epilepsy treatments. Neither do we know if it is more or less safe than other epilepsy treatments” (<https://www.epilepsy.org.uk/info/treatment/cannabis-based-treatments>) |
